# Cancer: Untethering Mitochondria from the Endoplasmic Reticulum?

**DOI:** 10.3389/fonc.2017.00105

**Published:** 2017-05-26

**Authors:** Maria Sol Herrera-Cruz, Thomas Simmen

**Affiliations:** ^1^Faculty of Medicine and Dentistry, Department of Cell Biology, University of Alberta, Edmonton, AB, Canada

**Keywords:** mitochondria-associated membrane, mitochondria-endoplasmic reticulum contacts, mitofusin-2, metabolism, oncoprotein, tumor suppressor

## Abstract

Following the discovery of the mitochondria-associated membrane (MAM) as a hub for lipid metabolism in 1990 and its description as one of the first examples for membrane contact sites at the turn of the century, the past decade has seen the emergence of this structure as a potential regulator of cancer growth and metabolism. The mechanistic basis for this hypothesis is that the MAM accommodates flux of Ca^2+^ from the endoplasmic reticulum (ER) to mitochondria. This flux then determines mitochondrial ATP production, known to be low in many tumors as part of the Warburg effect. However, low mitochondrial Ca^2+^ flux also reduces the propensity of tumor cells to undergo apoptosis, another cancer hallmark. Numerous regulators of this flux have been recently identified as MAM proteins. Not surprisingly, many fall into the groups of tumor suppressors and oncogenes. Given the important role that the MAM could play in cancer, it is expected that proteins mediating its formation are particularly implicated in tumorigenesis. Examples for such proteins are mitofusin-2 and phosphofurin acidic cluster sorting protein 2 that likely act as tumor suppressors. This review discusses how these proteins that mediate or regulate ER–mitochondria tethering are (or are not) promoting or inhibiting tumorigenesis. The emerging picture of MAMs in cancer seems to indicate that in addition to the downregulation of mitochondrial Ca^2+^ import, MAM defects are but one way how cancer cells control mitochondria metabolism and apoptosis.

## Introduction: Disruption of Mitochondrial Metabolism in Cancer

Lost control of mitochondria metabolism is a central cancer hallmark (1), although not all tumors are characterized by this property. As a consequence, cancer cells frequently rewire their metabolism to rely on glucose even in the presence of oxygen and, thus, reduce their reliance on mitochondria (2). In parallel, tumor cells exhibiting this so-called Warburg phenotype must increase their glycolytic capacity.

Multiple cancer signaling pathways are associated with the glycolytic signature of cancer. For instance, the excessive growth of many solid tumors results in large portions of the tumor mass becoming hypoxic, which subsequently induces production of key glycolysis enzymes *via* the HIF1α transcription factor (3), including glucose transporters (e.g., GLUT1) or glycolytic enzymes (e.g., phosphofructokinase) (4). Upon their induction, these enzymes shift energy generation away from mitochondria toward glycolysis and glutaminolysis (5–8). This allows tumor cells to accumulate more biomass through increased uptake and metabolism of glucose (2, 9–13). In parallel, while glycolysis only produces two ATP molecules per glucose molecule, compared to 36 molecules of ATP from the complete oxidation of glucose within mitochondria, glycolysis can still result in higher energy production due to speedier progression of this pathway and higher ATP production per time rate (14). Increased ATP consumption could further accelerate this pathway and could result in almost 100 times faster ATP generation than oxidative phosphorylation (15). Under these conditions, glucose is converted into lactate by conversion of pyruvate through lactate dehydrogenase (LDH) (16, 17). When LDH produces this glycolysis end product, it also replenishes NAD^+^ levels, which act to make the cytosol more oxidizing (18, 19). In tumor cells, however, lactate can also be shuttled to mitochondria, where it can be metabolized to synthesize lipids (20). Secreted leftover lactate contributes to the altered tumor microenvironment by lowering the extracellular pH, activation of the VEGF signaling pathway (21), and driving cell migration (22), to name but a few consequences (23). Together, the increased presence of lactate caused by tumor metabolism critically manipulates multiple metabolic pathways and cell biological mechanisms.

In parallel, HIF1α can also achieve another characteristic of the Warburg phenotype: the repression of oxidative phosphorylation by cooperating with c-Myc to drive transactivation of pyruvate dehydrogenase kinase 1 (PDK1) and its relatives (24, 25). The induction of this enzyme not only directly reduces mitochondrial oxygen consumption but also further promotes glycolysis by decreasing pyruvate flow to mitochondria, while increasing its conversion to lactate (24, 25). Therefore, the inhibition of PDK1 and related kinases by RNAi or dichloroacetate can potentially rescue some of the metabolic changes in tumor tissue (26).

While it was clear for a long time that cancer mitochondria make less ATP, it had initially been questioned whether the reason for this defect is found within the proteins making up the electron transport chain within mitochondria (27). However, many types of cancer result in a relative depletion of mtDNA, when compared to neighboring tissue (28), as one would expect given the important links between mitochondrial metabolism and cancer. Moreover, numerous mitochondrial enzymes encoded by nuclear or mitochondrial DNA show mutations (29). Specifically, mutations in mtDNA can indirectly affect enzymes of the Krebs cycle, including fumarate hydratase (30), and isocitrate dehydrogenase (31). Moreover, mutations in nuclear-encoded succinate dehydrogenase can by themselves cause paraganglioma (32, 33), potentially from increased ROS production within mitochondria that leads to oxidative damage and eventually transformation (34). Somatic mutations of mtDNA have been discovered in a wide variety of cancers, including colorectal, ovarian, renal, and lung cancers (35–38). Moreover, the depletion of mtDNA by itself can act as tumorigenic *in vitro* as well as *in vivo* (39). Such a loss of mtDNA, as well as mitochondrial mass, can be caused, for instance, by mutations of p53 (40). While an outright loss of mtDNA can sometimes paradoxically reduce mitochondrial ROS production due to arrest of oxidative phosphorylation (40), cancer tissue is normally characterized by increased levels of ROS, due to the rapid growth of tumors (41). Therefore, more frequently, mtDNA mutations accelerate tumor progression *via* increased ROS production that leads to further mutations within nuclear and mitochondrial DNA (42). This sets a dangerous cycle in motion that further increases the level of oxidative stress (43, 44). Accordingly, the type II diabetes drug metformin, which reduces both ROS and inhibits complex I (45, 46), reduces the risk of developing cancer (47, 48). This finding suggests that regulation of mitochondrial ROS production is an important point of intervention for the treatment of cancer. In addition, it is also clear that proteins regulating the progression of mitochondrial oxidative phosphorylation and, thus, production of mitochondrial ROS must be found on the list of tumor suppressors and oncoproteins.

## Oncoproteins and Tumor Suppressors Use the Endoplasmic Reticulum (ER) as a Platform to Control Mitochondria

Recent progress has determined that besides proteins mediating oxidative phosphorylation themselves, regulatory proteins outside mitochondria could determine mitochondrial ROS and tumorigenesis. An attractive location to execute such a function is the mitochondria-associated membrane (MAM) (49). This intracellular signaling hub houses Ca^2+^ exchange between the ER and mitochondria that is required for mitochondrial dehydrogenases and, thus, oxidative phosphorylation (49). Accordingly, cells with blocked active Ca^2+^ release from the ER produce less than half of their normal amount of mitochondrial ATP (50). A need for ER–mitochondria cross talk to fully engage cellular metabolism and energy production had been anticipated in early studies of Bernhard and Rouiller on the regenerating liver, where ER and mitochondria form close appositions in a fasting–feeding-dependent manner (51, 52). Today, altered Ca^2+^ signaling at the MAM is recognized as a hallmark of cancer cells that shifts their metabolism to glycolysis and increases their resistance to cell death (53).

Early studies had identified the MAM as a lipid synthesis platform, where phosphatidylethanolamine (PE) production requires the apposition between the ER and mitochondria (54–56). It is, therefore, not surprising that MAM lipid enzymatic activities are essential for normal Ca^2+^ signaling (57). Critically, the MAM represents a detergent-resistant membrane that forms a locally cholesterol-enriched raft (58, 59). This structure is enriched in the sigma-1 receptor (60), the ER prohibitin-related proteins erlin-1 and erlin-2 (61), the ubiquitin ligase gp78 (62), and the ER oxidoreductase TMX1 (63). Therefore, one way how tumor cells could silence mitochondria metabolism and apoptotic signaling would be by altering ER lipid domain formation that could disrupt normal MAM rafts.

Consistent with such a possibility, a variety of lipid-interfering strategies are currently in development to trigger ER stress-related apoptosis in cancer cells and have been reviewed recently (64). This idea is based on findings that show that cholesterol loading of the ER leads to ER stress and subsequent apoptosis of a variety of cell types (65–67). Mechanistically, this excess cholesterol efficiently blocks ER sarco/ER Ca^2+^-ATPase (SERCA) that pumps Ca^2+^ into the ER, thus resulting in the transfer of Ca^2+^ to mitochondria (68). Similarly, ER lipid saturation, achieved by elevated phosphatidylcholine over PE (69) or palmitate (70), activates the ER stress response and apoptosis *via* the inhibition of SERCA.

However, these mechanisms might all represent a drastic, artificial phenotype that cannot be exploited for cancer therapy and does not operate in the same way within cancer cells. Moreover, their potential links between cancer cell biology and MAM-related mechanisms are currently obscure. Even when restricting to studies on cholesterol and tumor cell function and survival, no clear picture emerges. For example, mitochondria of cancer cells are more susceptible to increases in cholesterol, which tend to trigger ER stress and apoptosis in this system more readily than in normal cells (67, 71). However, cholesterol-lowering drugs such as statins also trigger mitochondria-based apoptosis (72), possibly because cancer mitochondria operate with 2- to 10-fold more cholesterol than mitochondria of normal cells (73). Cholesterol- and PE-rich mitochondria also provide more resistance to Bax-mediated membrane permeabilization (74, 75). We must conclude that cancer cells might be influenced by cholesterol and that altering the lipid balance of cancer cells could affect their MAM rafts, but that a clear outcome of such interventions within cancer cells has not yet emerged. A potential explanation for these observations is that lipid storage at an early stage of cancer changes to lipolysis accompanied by increased cholesterol synthesis in advanced cancer (76, 77). In the context of this review, these observations suggest that the lipid- and cholesterol-dependent structure that is the MAM may undergo tumor stage-specific changes.

Nevertheless, consistent with altered lipid and cholesterol content of cancer mitochondria (73), ER–mitochondria tethering might be different at least in a subset of cancers. This hypothesis has been put forward over 60 years ago by Howatson and Ham (78), who observed reduced numbers of mitochondria and of ER–mitochondria contacts in liver cancer. These observations anticipated follow-up observations, which also detected lower amounts of mitochondria in tumor tissue in multiple instances (79–81). Moreover, despite (or maybe because of) their potentially increased distance from intracellular Ca^2+^ sources, some tumor cell mitochondria have an increased Ca^2+^ uptake capacity compared to mitochondria from normal tissue when examined as isolated entities *in vitro* (82, 83). This property is also reflected in the upregulation of mitochondrial Ca^2+^ uniporters (MCUs) in breast cancer cells (84). Potentially, however, MCU is also a target of miR-25 that can reduce its amount in cancer cells (85). While these findings suggest more research is needed to assess the role of MCU in cancer, they also demonstrate that tumor mitochondria have different Ca^2+^ handling. Moreover, the end result of both observations for cancer mitochondria could actually be the same. While some tumor mitochondria may import less Ca^2+^ *via* reduced amounts of MCU, others may show increased Ca^2+^ uptake capacity from a compensation for decreased Ca^2+^ availability in the tumor cell, maybe due to defective ER–mitochondria tethering. Generally speaking, these findings also indicate that cancer cells undertake massive remodeling of upstream signaling mechanisms that result in reduced mitochondrial ATP output in cancer cells, as postulated by Warburg (2). Importantly, as an end result, this remodeling may very well end up making the residual power generation within mitochondria essential (86).

**Figure 1:**
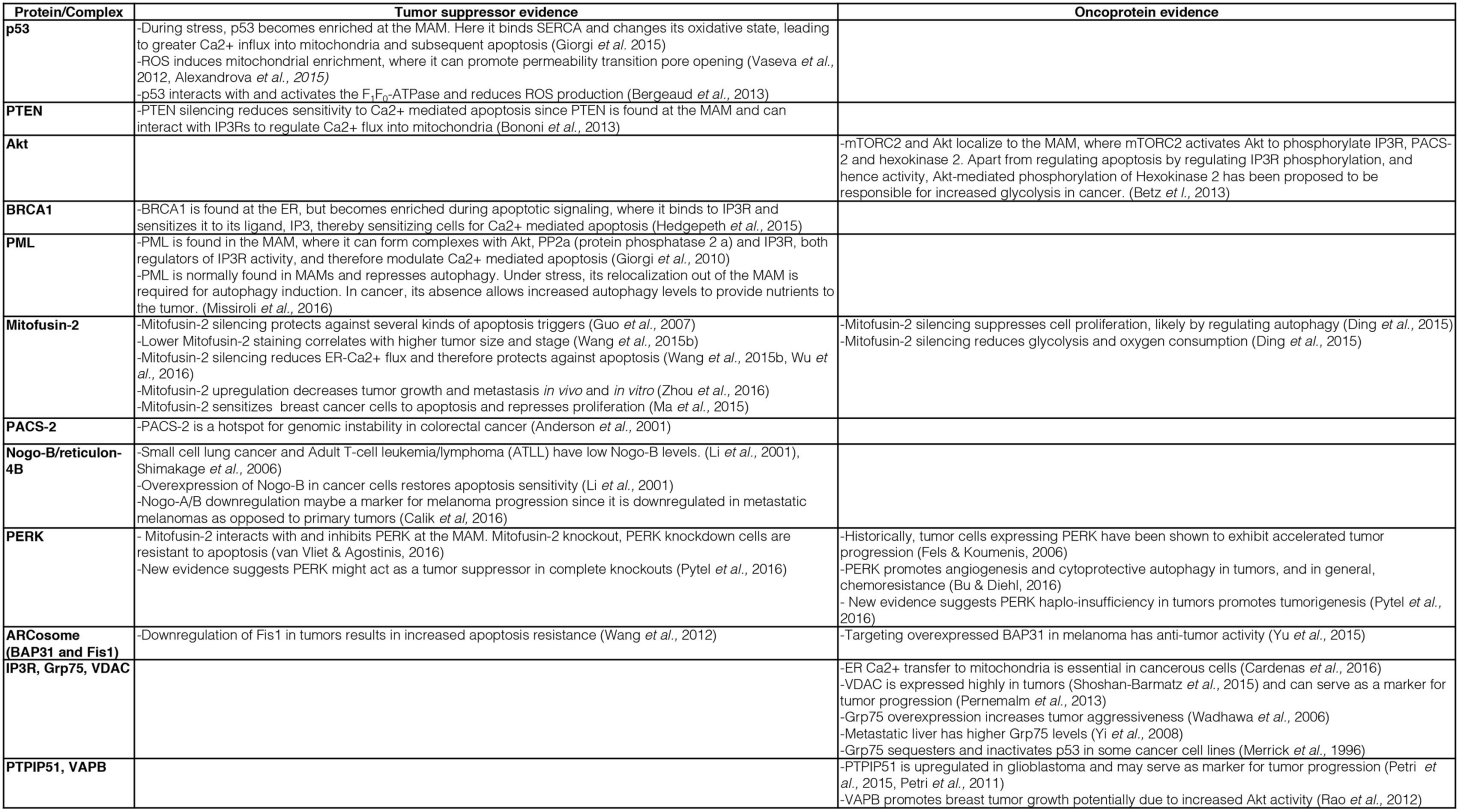
**Description of key evidence on the role of mitochondria-associated membrane (MAM) tethering factors as tumor suppressors or oncoproteins**.

Consistent with such a modulated ER–mitochondria Ca^2+^ flux, MAM-localized oncoproteins and tumor suppressors indeed interact with Ca^2+^ handling proteins and modulate their activity. Consistent with this hypothesis, the tumor suppressors and oncoproteins p53 (87), the phosphatase tensin homolog (PTEN) (88), the kinase Akt (89), breast/ovarian cancer susceptibility gene 1 (BRCA1) (90), and the promyelocytic leukemia (PML) protein (91, 92) all localize to mitochondria or to ER–mitochondria contacts. Here, they exert their cancer-regulating activities by interacting with Ca^2+^-handling proteins and either boost ER–mitochondria Ca^2+^ flux (tumor suppressors) or inhibit it (oncoproteins), for details see Figure 1.

For instance, p53 is enriched on the MAM, where it interacts with the ER Ca^2+^ pump SERCA and promotes ER–mitochondria Ca^2+^ cross talk not only to induce apoptosis but also to promote ATP production needed within the ER (93). Thus, the tumor suppressor p53 acts to beneficially facilitate existing mechanisms and, thus, overall improves mitochondrial functioning. In addition, it appears that these p53 functions depend on mitochondrial ROS production, since ROS induce p53 enrichment on mitochondria, where it can promote the opening of the permeability transition pore (PTP) (94) upon a stabilizing interaction with Hsp90 (95). This finding provides another connection between mitochondrial Ca^2+^ signaling, oxidative phosphorylation, and p53, but the interaction of this tumor suppressor with the mitochondrial proton gradient and Ca^2+^ signaling goes further. p53 can also influence mitochondria function *via* direct interaction with the F_1_F_0_-ATPase to promote respiration and reduce ROS production (96). Interestingly, this function could directly link p53 to the control of mitochondrial permeability transition, since the F_1_F_0_-ATPase or parts of it are the most likely candidates for forming the mitochondrial PTP (97–99). Given that hexokinase II localizes to the PTP (100), from where it increases the use of glucose (101), p53 and hexokinase II may oppose each other in the control of tumor cell growth, as is indeed the case in castration-resistant prostate cancer (102). Interestingly, hexokinase II binding to voltage-dependent anion channel (VDAC) increases in parallel with cholesterol loading of mitochondria, thus providing additional evidence that cancer-associated alterations of MAM and mitochondria properties shift cellular energy generation to glycolysis (73). All of these MAM-associated functions depict p53 as a factor that would beneficially control mitochondrial oxidative phosphorylation: not only as a gatekeeper, which would promote ER–mitochondria Ca^2+^ flux, but also as a chaperone, which can make mitochondrial ATP production more efficient and which can arrest mitochondrial ATP production in the case of excessive ROS production.

**Figure 2:**
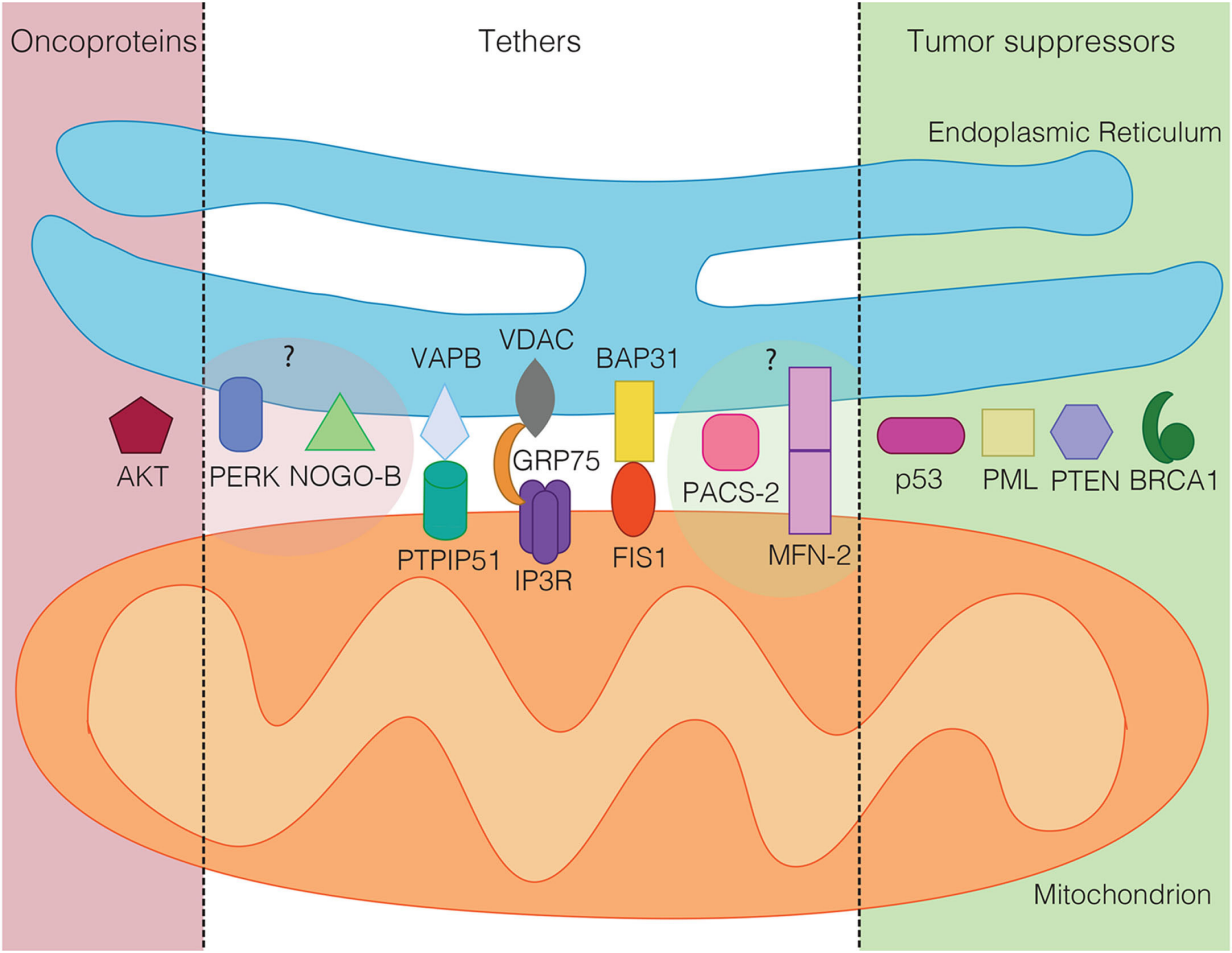
**Tumor suppressors (green) and oncoproteins (red) of the mitochondria-associated membrane grouped according to their demonstrated (dark shaded) or suspected role in cancer (light shaded)**. In the middle, known mammalian tethering regulators or protein complexes, whose role in cancer is ambiguous or unclear.

An important question that cell biologists are currently trying to answer is whether other mitochondrial regulatory proteins could fulfill similar roles to the ones described above for p53, PTEN, Akt, BRCA1, and PML. Given the characteristic mechanisms that these proteins use to influence mitochondrial metabolism and apoptosis regulation, such proteins should influence mitochondrial ROS and ATP production, likely *via* influencing the availability of Ca^2+^ within mitochondria. While recent reviews have provided outstanding global overviews of this hypothesis that we recommend the reader to consult as well (53, 103, 104), our review will specialize on the most central subcategory of proteins regulating the availability of Ca^2+^ within mitochondria. These are the proteinaceous tethers between the ER and mitochondria. While the identity of such proteinaceous tethers is currently much better understood in the yeast model system (105), where the ER–mitochondria encounter structure (ERMES) and ER membrane protein complex (EMC) are known or implicated in tethering the two organelles (106, 107), respectively, numerous proteins have been implicated in the formation or regulation of ER–mitochondria tethers in human cells (108). We will discuss these tether protein complexes as well as tethering regulators below. The current knowledge about ER–mitochondria tethers in cancer is summarized in Figures 1 and 2.

### Mitofusin-2

Mitofusins are a pair of GTPases that promote mitochondrial fusion (109). Mitofusins also determine ER–mitochondria apposition through a variety of proposed mechanisms. The most recent findings about their role for ER–mitochondria contacts suggest that they determine the outer mitochondrial membrane (OMM) protein composition (110). Through this function, mitofusins determine the surface properties of mitochondria, which could impact the interaction of mitochondria with the ER. Indeed, confirming this hypothesis, the expression balance between the two mitofusins regulates the relative apposition between mitochondria and the rough and smooth ER (rER/sER). Specifically, mitofusin-1 appears to inhibit the formation of sER–mitochondria contacts, whereas mitofusin-2 appears to interfere with the formation of rER–mitochondria contacts in cells with increased mitofusin-1 levels (111, 112). Currently, it is unclear whether this effect is *via* a direct regulation of contact formation between the two subpopulations of the ER with mitochondria, or whether the influence of the mitofusins on mitochondrial OMM proteins could explain these findings. In the latter scenario, protein subdomains on the OMM could mediate contact formation preferentially with the rER or sER. Regardless, these two findings clearly indicate that mitofusins determine the interaction between the ER and mitochondria. However, the exact role and the actual consequences of the mitofusins for this interorganellar interaction are currently being hotly debated.

In the case of mitofusin-2, the role for ER–mitochondria tethering extends beyond the regulation of the proportion between sER and rER–mitochondria contact formation, since the Scorrano lab had identified this mitochondrial GTPase as globally critical for MAM formation in mammalian cells (113). This role of mitofusin-2 in ER–mitochondria tethering was identified *via* a loss of FRET signal from two distinct ER–mitochondria proximity indicator probes (114, 115), by a reduction of fluorescence signal overlap between mitochondrial RFP and ER YFP, by a reduced mitochondrial uptake of IP_3_R-released ER Ca^2+^ (113), by reduced numbers of ER tubules in the proximity of mitochondria on electron micrographs (116), by increased resistance to ER stress-mediated apoptosis (117), and by a reduction of a coefficient that measures the extent of close ER contacts relative to the total mitochondria surface (115). However, these results did not unequivocally determine whether mitofusin-2 is an actual tether or whether it simply controls tethering. Moreover, despite these multiple results from many experimental approaches suggesting mitofusin-2 is an ER–mitochondria tether, these findings have been challenged by studies that measured the actual distance of ER–mitochondria contacts *via* electron microscopy and found a decrease in ER–mitochondria contacts in Mfn2^−/−^ cells (118, 119). Moreover, etoposide- and ceramide-mediated apoptosis proceeds faster in Mfn2 knockdown cells (119). As discussed by others and us recently (105, 108), multiple hypotheses could explain these discrepant findings. In our opinion, a compelling observation has been made recently by the Scorrano lab, which identified cellular culture conditions as critical for the role of mitofusin-2 for the formation of MAMs (115). This explanation would be an extension of the observations that mitofusin-2 knockout cells exhibit ER stress and that ER stress increases MAM contact formation (120, 121). Regardless of these outstanding questions, it is undisputed by all researchers of the field that mitofusins, and particularly mitofusin-2, are important regulators of MAM contacts. However, further research will have to determine the biogenesis and consequences of the reported phenotypes.

A role in ER–mitochondria contact formation raises the possibility that mitofusin-2 could also play a role as an oncoprotein or tumor suppressor. Since normal apoptosis progression requires a functional MAM, understanding its role in cancer may provide clues as to what function mitofusin-2 performs for the MAM. Indeed, and consistent with a role of mitofusin-2 as a MAM promoter, cancer cells with high levels of mitofusin-2 are more susceptible for apoptosis and more competent for ER–mitochondria Ca^2+^ flux (122–124). Further demonstrating the tumor-suppressive role of mitofusin-2, a panel of hepatocellular carcinoma (HCC) showed significant downregulation of mitofusin-2 and correlated with worse overall survival (125). Accordingly, mitofusin-2 mRNA is targeted by miR-761 in HCC tissues. The upregulation of mitofusin-2 *via* inhibiting miR-761 decreased tumor growth and metastasis both *in vivo* and *in vitro* (126). Similar findings have been reported from breast cancer cells, where the ectopic expression of mitofusin-2 leads to pro-apoptotic and antiproliferative signaling (127). Consistent with these findings, mitofusin-2 knockdown leads to reduced respiration, presumably due to blocked ER–mitochondria Ca^2+^ flux, but also reduces glycolysis, thus reducing overall ATP levels in HeLa cells (128). Together, these observations indicate that mitofusin-2 is a factor in cancer that typically results as reduced or absent in the cancer scenario (Figures 1 and 2). While some of the findings may turn out to be cell-type specific, these findings are more consistent with a role of mitofusin-2 as a MAM promoter and a tumor suppressor.

### Phosphofurin Acidic Cluster Sorting Protein 2 (PACS-2)

About 10 years ago, the cytosolic PACS-2 was identified as a homolog of the previously identified PACS-1 (129, 130). Unlike its closely related sister protein PACS-1 that regulates trafficking at the level of the trans-Golgi network and endosomes (131), PACS-2 determines the interaction between the ER and mitochondria, consistent with its partial localization to these organelles (130). Besides targeting of Bid to mitochondria and other functions described elsewhere (132), PACS-2 is required for the proper formation of the MAM (57, 133).

Here, PACS-2 acts as a MAM tethering regulator, but likely does not function as a MAM tether on its own. PACS-2 knockdown or knockout, nevertheless, interferes with several key MAM functions. For instance, PACS-2 knockdown detaches the ER from mitochondria, as seen by light and electron microscopy (130). This and other activities of PACS-2 depend on its serine 437 residue, which promotes active PACS-2 (134). Moreover, the phosphorylation of this site by Akt is a prerequisite to maintain MAM formation and is downstream of mammalian target of rapamycin complex 2 (mTORC2) (89). mTORC2/Akt-mediated phosphorylation of PACS-2 maintains proper Ca^2+^ availability for mitochondria, needed for mitochondria metabolism (50), but also apoptosis progression (135). From this insight, it makes, perfect sense that in a cancer scenario, PACS-2 is a hot spot of chromosome instability, as indeed observed in colorectal cancer (136), possibly in a stage-dependent manner (137). Therefore, similar to the better-characterized mitofusin-2, it is expected that PACS-2 acts as a tumor suppressor (Figures 1 and 2), whose absence would be indeed expected to lead to ER–mitochondria uncoupling, but this has not been determined at this point. No information is currently available about cancer-associated mutations in PACS-2, but it is clear that the regulatory serine 437 residue would correspond to a prime candidate.

### Nogo-B/Reticulon-4B

Like mitofusin-2 and PACS-2, Nogo-B/reticulon-4B is a structural regulator of the ER, promoting the formation of tubular ER (138). Compared to the highly related Nogo-A that is restricted to neuronal cells, Nogo-B is expressed ubiquitously (139). Upon overexpression of this protein, the proportion of tubular ER increases over sheet-like ER (138). A Nogo-B overexpression could occur, for instance, during ER stress or hypoxia that leads to increased reticulon-4 expression dependent on the ER transcription factor ATF6 (140). Interestingly in the context of this review, increased Nogo-B expression associated with hypoxia increases the distance between the ER and mitochondria, suggesting that Nogo-B acts as an inhibitor of ER–mitochondria tethering (141). Nogo-B is not the only reticulon that localizes to the MAM and whose overexpression modulates ER–mitochondria contact formation: the same has been reported for reticulon-1C, although its activity seems to be opposite (142).

Again, like in the case of mitofusin-2 and PACS-2, the question arises as to what is the functional basis of a role for Nogo-B in regulating the apposition between the ER and mitochondria. It appears that a common pattern is emerging, where ER- and mitochondria-associated factors that determine their respective membrane composition or shape also increase or decrease organellar apposition. This is again confirmed upon knockout of Nogo-B. In this scenario, ER tubulation is lost and the diameter of ER structures increases (143). Apparently contradicting a role as a MAM inhibitor, cells lacking Nogo-B are resistant to apoptosis, which normally depends on ER–mitochondria Ca^2+^ flux. While this finding could suggest that the role of Nogo-B is less clear than anticipated, this effect could also depend on a role of Nogo-B on the apposition between the ER and the plasma membrane: Nogo-B-deficient cells exhibit decreased store-operated Ca^2+^ entry, which suggests that this reticulon acts to increase contacts between the ER and the plasma membrane (143). This observation raises the important issue that ER tethering factors could promote apposition in the case of contacts with one organelle, but decrease apposition in the case of contacts with other organelles.

If our hypothesis were correct that ER–mitochondria tethering antagonizes tumor progression, then we would expect to find expression of a MAM-inhibitory Nogo-B to be high in cancer. However, the first paper linking Nogo-B to cancer found this to be the opposite (144): ectopic expression of Nogo-B restores apoptosis susceptibility in cancer cells, and small cell lung cancer was found to exhibit low levels of Nogo-B. Similarly, low levels of Nogo-B were found in leukemia and lymphoma (145), as well as in metastatic malignant melanoma (146). Along these lines, it is possible to see the effect of Ras transformation that results in cleavage of Nogo-B as a disruption of its MAM-regulating activities (147). Together, these observations are more consistent with a role of Nogo-B in promoting cell death.

While these findings may suggest that MAM tethering cannot be unequivocally tied to tumor suppression, additional cancer-relevant functions could complicate the role in cancer for Nogo-B. Besides the previously mentioned role for ER–plasma membrane apposition by Nogo-B, such functions have indeed been detected in the case of Nogo-A, the Nogo form expressed in the central nervous system that acts to promote MAM formation. In addition to regulating the MAM, Nogo-A downregulates Rho signaling in neuronal cells and thus inhibits migration of glioma cells (148). Nogo-A also stabilizes the receptor tyrosine kinases ErbB3 and ErbB4 through the sequestration of their ubiquitin ligase Nrdp1 within ER tubules. This then results in an increase in proliferative signaling upon Nogo-A overexpression (149). Such secondary functions likely preclude a clear, logical connection of Nogo-B between its published role in MAM suppression and its activities as a tumor suppressor as well.

### Protein Kinase RNA-Like ER Kinase (PERK)

A more recently discovered tethering factor is the ER kinase PERK (150). While PERK is more commonly known as the kinase that phosphorylates eukaryotic initiation factor 2α and thus blocks translation of ER proteins under ER stress conditions (151), PERK also localizes to the MAM, where it promotes the apposition between the ER and mitochondria. Accordingly, PERK knockout cells exhibit a MAM that is less tight and show resistance to apoptosis inducers (152). Interestingly, these functions of PERK at the MAM are accompanied by its interaction with mitofusin-2 (117). A general role of PERK in the functioning of membrane contact sites is confirmed by its role in the formation of ER contacts with the plasma membrane (153).

From these functions, and if restricting a cancer role to its function on the MAM, we would predict that PERK, like PACS-2 and mitofusin-2, should act as a tumor suppressor. However, historically, PERK-expressing tumor cells have been found to have a growth advantage (154). This finding is based on the role of PERK in the unfolded protein response, where it protects cells against oxidative stress originating from the ER (155). Despite the induction of the pro-apoptotic transcription factor CHOP, PERK tends to elicit a tumor-promoting function due to its role in increasing angiogenesis as well as resistance to chemotherapy (156). Hence, the picture of PERK in cancer might be complex. Consistent with this idea and as expected from its ambiguous role in ER stress and MAM tethering, more recently it has become clear that PERK can promote both tumor progression and suppression (157).

### Multimeric Mammalian MAM Tethering Complexes

While the yeast model system has shown that its MAM relies on multimeric, ER- and mitochondria-localized protein complexes [ERMES and EMC (105)], these complexes either do not exist in mammalian cells (ERMES) or their functioning in tethering is currently unknown (EMC). Nevertheless, the set of proteins mediating the tethering of the ER to mitochondria is expected to comprise multimeric protein complexes that localize to both the ER and mitochondria in mammalian cells as well. Indeed, a couple of multimeric MAM tethering complexes have been proposed to exist over the past decade. One such protein complex is the ARCosome that is formed when ER-localized BAP31 interacts with mitochondrial Fis1 (158). Interestingly, the ARCosome undergoes modulation upon cell stress, which results in its association with caspase-8. This interaction alters the function of the ARCosome, which then becomes involved in mitochondrial fission through formation of the p20 fragment of BAP31 (159). This suggests the ARCosome could be central in pro-apoptotic roles of the MAM, suggesting that cancer is characterized by its absence or disruption.

However, not much is known about components of the ARCosome and cancer. A recent publication suggests that BAP31 is overexpressed in malignant melanoma (160). While this finding apparently contradicts our expectations, it might result in altered pro-apoptotic signaling of the ARCosome. More aligned with the idea that the ARCosome would suppress tumor growth, miR-484 downregulates Fis1 in cancer, associated with increased cancer resistance (161).

Another ER–mitochondria protein complex consists in the association between IP_3_Rs, the voltage-gated anion channel (VDAC), and the OMM chaperone Grp75 (162). Within this complex, VDAC (163) and Grp75 (162) act to boost ER–mitochondria Ca^2+^ flux. However, the exact importance of this complex for the formation and maintenance of the MAM is not known, since IP_3_R triple knockout cells do not show an altered MAM (121). Moreover, it is not known whether deletion or overexpression of any member of this complex modulates MAM formation. Nevertheless, the transfer of Ca^2+^ from the ER to mitochondria accommodated by IP_3_Rs and VDAC is typically low in cancer cells, but essential (164). Not surprisingly, the members of this protein complex exhibit multiple connections to cancer, and all are important regulators of cell survival and cell death.

VDAC is an important control point not only for the influx of Ca^2+^ into mitochondria but also for the efflux of pro-apoptotic molecules and, thus, controls both mitochondria metabolism and cell death. Typically, VDAC is highly expressed in tumor tissue (165), and its expression level has been proposed to correlate so much with poor prognosis to be a candidate biomarker (166). Grp75 is also called mortalin, due to its antiproliferative effects (167). In cancer, however, Grp75 appears to act tumor-promoting, since its expression increases upon liver cancer metastasis (168) and overexpression of Grp75 increases the aggressiveness of a variety of tumor cell lines (169). Here, like in the case of VDAC and PERK, additional, MAM-unrelated functions may lead to a complex readout of the role of Grp75 in cancer. One such example may be that Grp75 can sequester and inactivate p53 (170).

A more recently described ER–mitochondria tethering complex is based on the OMM protein PTPIP51 and the ER vesicle-associated membrane protein-associated protein B (VAPB) that spans the ER membrane. Indicative of its role in ER–mitochondria tethering, depleting its components disrupts mitochondrial Ca^2+^ import (171). PTPIP51 is known to be upregulated in glioblastoma, a role which may depend on the function of PTPIP51 as a promoter of growth factor signaling (172, 173). Similarly, VAPB also has a growth-stimulatory activity of tumor tissue that might be tied to increased activity of Akt when VAPB is highly expressed (174). The oncoproteins TDP-43 (175) and fused in sarcoma (FUS) inhibit the PTPIP51–VAPB complex (176), again suggesting that the proteins of this complex generally act to accelerate tumor growth, albeit not necessarily through their roles at the MAM.

Together, it appears that the currently known multimeric protein complexes of the MAM have unclear roles for tumorigenesis that appear not always linked to their functions as MAM tethers. But given their rather recent identification as such tethers, and the many open questions about this biological role, such statements should not be considered as final.

## Conclusion

Research from the past decade has identified the MAM as a potentially central regulator of tumor cell metabolism, as exemplified by the presence of critical tumor suppressors and oncoproteins on this structure. Moreover, findings from our lab and others have shown that MAM proteins such as the oxidoreductase TMX1 indeed can determine the balance between tumor cell glycolysis and oxidative phosphorylation (89, 177). From these findings and early insights (78), we could postulate that in particular solid, glycolytic tumor tissue is frequently characterized by a loss of normal MAM architecture and formation. Further research will have to determine whether this is indeed the case for a majority of cancer types.

There is no doubt that proteins forming connections between the ER and mitochondria are differentially expressed in tumor tissue, as shown by numerous examples mentioned in this review. Additionally, many of these proteins are multifunctional, leading to complex significance for tumor growth that is not limited to the maintenance of the MAM. Therefore, with the exception of mitofusin-2 and PACS-2, most MAM tethering regulators show no clear association with the progression of cancer or no logical connection of their expression pattern to their role as MAM tethers. One reason for this lack of a clear link could be the often multifunctional properties of MAM regulatory proteins. Another reason is that the bigger picture of changes at the MAM may impact the survival and proliferation of cancer cells in more ways than one.

Given the field is rapidly developing, and the exact roles of MAM regulators are still evolving, such connections may solidify in the coming years. Additionally, since the entire set of MAM tethers in mammalian cells is almost certainly incomplete, new tethers may emerge that show better or cleaner association with tumorigenesis than the ones we currently know. Therefore, researchers studying the role of ER–mitochondria contacts in tumor cell metabolism and tumorigenesis are expected to read about further exciting findings in the near future that will identify more oncoproteins and tumor suppressors on this suborganellar domain of the ER.

## Author Contributions

TS wrote the manuscript. MH-C contributed to text sections, edited the text, and produced the figure and table for the manuscript.

## Conflict of Interest Statement

The research was conducted in the absence of any commercial or financial relationships that could be construed as a potential conflict of interest.

## References

[1] Hanahan D Weinberg RA Hallmarks of cancer: the next generation. Cell (2011) 144:646–74. 10.1016/j.cell.2011.02.013 21376230

[2] Warburg O On the origin of cancer cells. Science (1956) 123:309–14. 10.1126/science.123.3191.309 13298683

[3] Altenberg B Greulich KO. Genes of glycolysis are ubiquitously overexpressed in 24 cancer classes. Genomics (2004) 84:1014–20. 10.1016/j.ygeno.2004.08.010 15533718

[4] Zelzer E Levy Y Kahana C Shilo BZ Rubinstein M Cohen B. Insulin induces transcription of target genes through the hypoxia-inducible factor HIF-1alpha/ARNT. EMBO J (1998) 17:5085–94. 10.1093/emboj/17.17.5085 9724644 PMC1170836

[5] Ebert BL Firth JD Ratcliffe PJ. Hypoxia and mitochondrial inhibitors regulate expression of glucose transporter-1 via distinct Cis-acting sequences. J Biol Chem (1995) 270:29083–9. 10.1074/jbc.270.49.29083 7493931

[6] Ebert BL Gleadle JM O’Rourke JF Bartlett SM Poulton J Ratcliffe PJ. Isoenzyme-specific regulation of genes involved in energy metabolism by hypoxia: similarities with the regulation of erythropoietin. Biochem J (1996) 313(Pt 3):809–14. 10.1042/bj3130809 8611159 PMC1216982

[7] Lu CW Lin SC Chen KF Lai YY Tsai SJ. Induction of pyruvate dehydrogenase kinase-3 by hypoxia-inducible factor-1 promotes metabolic switch and drug resistance. J Biol Chem (2008) 283:28106–14. 10.1074/jbc.M803508200 18718909 PMC2661383

[8] Obach M Navarro-Sabate A Caro J Kong X Duran J Gomez M 6-phosphofructo-2-kinase (pfkfb3) gene promoter contains hypoxia-inducible factor-1 binding sites necessary for transactivation in response to hypoxia. J Biol Chem (2004) 279:53562–70. 10.1074/jbc.M406096200 15466858

[9] Curi R Newsholme P Newsholme EA. Metabolism of pyruvate by isolated rat mesenteric lymphocytes, lymphocyte mitochondria and isolated mouse macrophages. Biochem J (1988) 250:383–8. 10.1042/bj2500383 3128282 PMC1148867

[10] Eagle H Oyama VI Levy M Horton CL Fleischman R The growth response of mammalian cells in tissue culture to l-glutamine and l-glutamic acid. J Biol Chem (1956) 218:607–16. 13295214

[11] Kovacevic Z McGivan JD Mitochondrial metabolism of glutamine and glutamate and its physiological significance. Physiol Rev (1983) 63:547–605. 6132422 10.1152/physrev.1983.63.2.547

[12] Locasale JW Cantley LC. Metabolic flux and the regulation of mammalian cell growth. Cell Metab (2011) 14:443–51. 10.1016/j.cmet.2011.07.014 21982705 PMC3196640

[13] Lunt SY Vander Heiden MG. Aerobic glycolysis: meeting the metabolic requirements of cell proliferation. Annu Rev Cell Dev Biol (2011) 27:441–64. 10.1146/annurev-cellbio-092910-154237 21985671

[14] Pfeiffer T Schuster S Bonhoeffer S. Cooperation and competition in the evolution of ATP-producing pathways. Science (2001) 292:504–7. 10.1126/science.1058079 11283355

[15] Fang M Shen Z Huang S Zhao L Chen S Mak TW The ER UDPase ENTPD5 promotes protein N-glycosylation, the Warburg effect, and proliferation in the PTEN pathway. Cell (2010) 143:711–24. 10.1016/j.cell.2010.10.010 21074248

[16] Adeva-Andany M Lopez-Ojen M Funcasta-Calderon R Ameneiros-Rodriguez E Donapetry-Garcia C Vila-Altesor M Comprehensive review on lactate metabolism in human health. Mitochondrion (2014) 17:76–100. 10.1016/j.mito.2014.05.007 24929216

[17] Burk D Woods M Hunter J On the significance of glucolysis for cancer growth, with special reference to Morris rat hepatomas. J Natl Cancer Inst (1967) 38:839–63. 4381692

[18] Adeva M Gonzalez-Lucan M Seco M Donapetry C. Enzymes involved in L-lactate metabolism in humans. Mitochondrion (2013) 13:615–29. 10.1016/j.mito.2013.08.011 24029012

[19] LaReau RD Anderson VE. An inquiry into the source of stereospecificity of lactate dehydrogenase using substrate analogues and molecular modeling. Biochemistry (1992) 31:4174–80. 10.1021/bi00132a004 1567864

[20] Chen YJ Mahieu NG Huang X Singh M Crawford PA Johnson SL Lactate metabolism is associated with mammalian mitochondria. Nat Chem Biol (2016) 12:937–43. 10.1038/nchembio.2172 27618187 PMC5069139

[21] Beckert S Farrahi F Aslam RS Scheuenstuhl H Konigsrainer A Hussain MZ Lactate stimulates endothelial cell migration. Wound Repair Regen (2006) 14:321–4. 10.1111/j.1743-6109.2006.00127.x 16808811

[22] Goetze K Walenta S Ksiazkiewicz M Kunz-Schughart LA Mueller-Klieser W. Lactate enhances motility of tumor cells and inhibits monocyte migration and cytokine release. Int J Oncol (2011) 39:453–63. 10.3892/ijo.2011.1055 21617859

[23] Martinez-Outschoorn UE Peiris-Pages M Pestell RG Sotgia F Lisanti MP. Cancer metabolism: a therapeutic perspective. Nat Rev Clin Oncol (2017) 14:11–31. 10.1038/nrclinonc.2016.60 27141887

[24] Kim JW Tchernyshyov I Semenza GL Dang CV. HIF-1-mediated expression of pyruvate dehydrogenase kinase: a metabolic switch required for cellular adaptation to hypoxia. Cell Metab (2006) 3:177–85. 10.1016/j.cmet.2006.02.002 16517405

[25] Papandreou I Cairns RA Fontana L Lim AL Denko NC. HIF-1 mediates adaptation to hypoxia by actively downregulating mitochondrial oxygen consumption. Cell Metab (2006) 3:187–97. 10.1016/j.cmet.2006.01.012 16517406

[26] Sutendra G Michelakis ED. Pyruvate dehydrogenase kinase as a novel therapeutic target in oncology. Front Oncol (2013) 3:38. 10.3389/fonc.2013.00038 23471124 PMC3590642

[27] Weinhouse S The Warburg hypothesis fifty years later. Z Krebsforsch Klin Onkol Cancer Res Clin Oncol (1976) 87:115–26. 10.1007/BF00284370 136820

[28] Reznik E Miller ML Senbabaoglu Y Riaz N Sarungbam J Tickoo SK Mitochondrial DNA copy number variation across human cancers. Elife (2016) 5:e10769. 10.7554/eLife.10769 26901439 PMC4775221

[29] Gaude E Frezza C. Defects in mitochondrial metabolism and cancer. Cancer Metab (2014) 2:10. 10.1186/2049-3002-2-10 25057353 PMC4108232

[30] Tomlinson IP Alam NA Rowan AJ Barclay E Jaeger EE Kelsell D Germline mutations in FH predispose to dominantly inherited uterine fibroids, skin leiomyomata and papillary renal cell cancer. Nat Genet (2002) 30:406–10. 10.1038/ng849 11865300

[31] Yan H Parsons DW Jin G McLendon R Rasheed BA Yuan W IDH1 and IDH2 mutations in gliomas. N Engl J Med (2009) 360:765–73. 10.1056/NEJMoa0808710 19228619 PMC2820383

[32] Baysal BE Ferrell RE Willett-Brozick JE Lawrence EC Myssiorek D Bosch A Mutations in SDHD, a mitochondrial complex II gene, in hereditary paraganglioma. Science (2000) 287:848–51. 10.1126/science.287.5454.848 10657297

[33] Niemann S Muller U. Mutations in SDHC cause autosomal dominant paraganglioma, type 3. Nat Genet (2000) 26:268–70. 10.1038/81551 11062460

[34] Ishii T Yasuda K Akatsuka A Hino O Hartman PS Ishii N. A mutation in the SDHC gene of complex II increases oxidative stress, resulting in apoptosis and tumorigenesis. Cancer Res (2005) 65:203–9. 15665296

[35] Jin X Zhang J Gao Y Ding K Wang N Zhou D Relationship between mitochondrial DNA mutations and clinical characteristics in human lung cancer. Mitochondrion (2007) 7:347–53. 10.1016/j.mito.2007.06.003 17707697

[36] Liu VW Shi HH Cheung AN Chiu PM Leung TW Nagley P High incidence of somatic mitochondrial DNA mutations in human ovarian carcinomas. Cancer Res (2001) 61:5998–6001. 11507041

[37] Nagy A Wilhelm M Sukosd F Ljungberg B Kovacs G Somatic mitochondrial DNA mutations in human chromophobe renal cell carcinomas. Genes Chromosomes Cancer (2002) 35:256–60. 10.1002/gcc.10118 12353267

[38] Polyak K Li Y Zhu H Lengauer C Willson JK Markowitz SD Somatic mutations of the mitochondrial genome in human colorectal tumours. Nat Genet (1998) 20:291–3. 10.1038/3108 9806551

[39] Cairns RA Iqbal J Lemonnier F Kucuk C de Leval L Jais JP IDH2 mutations are frequent in angioimmunoblastic T-cell lymphoma. Blood (2012) 119:1901–3. 10.1182/blood-2011-11-391748 22215888 PMC3293643

[40] Lebedeva MA Eaton JS Shadel GS. Loss of p53 causes mitochondrial DNA depletion and altered mitochondrial reactive oxygen species homeostasis. Biochim Biophys Acta (2009) 1787:328–34. 10.1016/j.bbabio.2009.01.004 19413947 PMC2680458

[41] Laurent A Nicco C Chereau C Goulvestre C Alexandre J Alves A Controlling tumor growth by modulating endogenous production of reactive oxygen species. Cancer Res (2005) 65:948–56. 15705895

[42] Gupta-Elera G Garrett AR Robison RA O’Neill KL. The role of oxidative stress in prostate cancer. Eur J Cancer Prev (2012) 21:155–62. 10.1097/CEJ.0b013e32834a8002 21857523

[43] Arsova-Sarafinovska Z Eken A Matevska N Erdem O Sayal A Savaser A Increased oxidative/nitrosative stress and decreased antioxidant enzyme activities in prostate cancer. Clin Biochem (2009) 42:1228–35. 10.1016/j.clinbiochem.2009.05.009 19465015

[44] Sharifi N Hurt EM Thomas SB Farrar WL. Effects of manganese superoxide dismutase silencing on androgen receptor function and gene regulation: implications for castration-resistant prostate cancer. Clin Cancer Res (2008) 14:6073–80. 10.1158/1078-0432.CCR-08-0591 18829485 PMC2670581

[45] Buzzai M Jones RG Amaravadi RK Lum JJ DeBerardinis RJ Zhao F Systemic treatment with the antidiabetic drug metformin selectively impairs p53-deficient tumor cell growth. Cancer Res (2007) 67:6745–52. 10.1158/0008-5472.CAN-06-4447 17638885

[46] Viollet B Guigas B Sanz Garcia N Leclerc J Foretz M Andreelli F. Cellular and molecular mechanisms of metformin: an overview. Clin Sci (Lond) (2012) 122:253–70. 10.1042/CS20110386 22117616 PMC3398862

[47] Evans JM Donnelly LA Emslie-Smith AM Alessi DR Morris AD Metformin and reduced risk of cancer in diabetic patients. BMJ (2005) 330:1304–5. 10.1136/bmj.38415.708634.F7 15849206 PMC558205

[48] Libby G Donnelly LA Donnan PT Alessi DR Morris AD Evans JM. New users of metformin are at low risk of incident cancer: a cohort study among people with type 2 diabetes. Diabetes Care (2009) 32:1620–5. 10.2337/dc08-2175 19564453 PMC2732153

[49] Raturi A Simmen T. Where the endoplasmic reticulum and the mitochondrion tie the knot: the mitochondria-associated membrane (MAM). Biochim Biophys Acta (2013) 1833:213–24. 10.1016/j.bbamcr.2012.04.013 22575682

[50] Cardenas C Miller RA Smith I Bui T Molgo J Muller M Essential regulation of cell bioenergetics by constitutive InsP3 receptor Ca2+ transfer to mitochondria. Cell (2010) 142:270–83. 10.1016/j.cell.2010.06.007 20655468 PMC2911450

[51] Bernhard W Haguenau F Gautier A Oberling C [Submicroscopical structure of cytoplasmic basophils in the liver, pancreas and salivary gland; study of ultrafine slices by electron microscope]. Z Zellforsch Mikrosk Anat (1952) 37:281–300. 10.1007/BF00343816 13123257

[52] Bernhard W Rouiller C Close topographical relationship between mitochondria and ergastoplasm of liver cells in a definite phase of cellular activity. J Biophys Biochem Cytol (1956) 2:73–8. 10.1083/jcb.2.4.73 13357525 PMC2229714

[53] Bittremieux M Parys JB Pinton P Bultynck G. ER functions of oncogenes and tumor suppressors: modulators of intracellular Ca(2+) signaling. Biochim Biophys Acta (2016) 1863:1364–78. 10.1016/j.bbamcr.2016.01.002 26772784

[54] Rusinol AE Cui Z Chen MH Vance JE. A unique mitochondria-associated membrane fraction from rat liver has a high capacity for lipid synthesis and contains pre-Golgi secretory proteins including nascent lipoproteins. J Biol Chem (1994) 269:27494–502. 7961664

[55] Vance JE. Phospholipid synthesis in a membrane fraction associated with mitochondria. J Biol Chem (1990) 265:7248–56. 2332429

[56] Vance JE Newly made phosphatidylserine and phosphatidylethanolamine are preferentially translocated between rat liver mitochondria and endoplasmic reticulum. J Biol Chem (1991) 266:89–97. 1898727

[57] Vance JE. MAM (mitochondria-associated membranes) in mammalian cells: lipids and beyond. Biochim Biophys Acta (2014) 1841:595–609. 10.1016/j.bbalip.2013.11.014 24316057

[58] Area-Gomez E Del Carmen Lara Castillo M Tambini MD Guardia-Laguarta C de Groof AJ Madra M Upregulated function of mitochondria-associated ER membranes in Alzheimer disease. EMBO J (2012) 31:4106–23. 10.1038/emboj.2012.202 22892566 PMC3492725

[59] Hayashi T Fujimoto M. Detergent-resistant microdomains determine the localization of sigma-1 receptors to the endoplasmic reticulum-mitochondria junction. Mol Pharmacol (2010) 77:517–28. 10.1124/mol.109.062539 20053954 PMC2845942

[60] Hayashi T Su TP. Sigma-1 receptors (sigma(1) binding sites) form raft-like microdomains and target lipid droplets on the endoplasmic reticulum: roles in endoplasmic reticulum lipid compartmentalization and export. J Pharmacol Exp Ther (2003) 306:718–25. 10.1124/jpet.103.051284 12730355

[61] Browman DT Resek ME Zajchowski LD Robbins SM. Erlin-1 and erlin-2 are novel members of the prohibitin family of proteins that define lipid-raft-like domains of the ER. J Cell Sci (2006) 119:3149–60. 10.1242/jcs.03060 16835267

[62] Goetz JG Nabi IR. Interaction of the smooth endoplasmic reticulum and mitochondria. Biochem Soc Trans (2006) 34:370–3. 10.1042/BST0340370 16709164

[63] Lynes EM Bui M Yap MC Benson MD Schneider B Ellgaard L Palmitoylated TMX and calnexin target to the mitochondria-associated membrane. EMBO J (2012) 31:457–70. 10.1038/emboj.2011.384 22045338 PMC3261551

[64] Beloribi-Djefaflia S Vasseur S Guillaumond F. Lipid metabolic reprogramming in cancer cells. Oncogenesis (2016) 5:e189. 10.1038/oncsis.2015.49 26807644 PMC4728678

[65] Feng B Yao PM Li Y Devlin CM Zhang D Harding HP The endoplasmic reticulum is the site of cholesterol-induced cytotoxicity in macrophages. Nat Cell Biol (2003) 5:781–92. 10.1038/ncb1035 12907943

[66] Lu X Liu J Hou F Liu Z Cao X Seo H Cholesterol induces pancreatic beta cell apoptosis through oxidative stress pathway. Cell Stress Chaperones (2011) 16:539–48. 10.1007/s12192-011-0265-7 21472505 PMC3156264

[67] Rios-Marco P Martin-Fernandez M Soria-Bretones I Rios A Carrasco MP Marco C. Alkylphospholipids deregulate cholesterol metabolism and induce cell-cycle arrest and autophagy in U-87 MG glioblastoma cells. Biochim Biophys Acta (2013) 1831:1322–34. 10.1016/j.bbalip.2013.05.004 23707264

[68] Li Y Ge M Ciani L Kuriakose G Westover EJ Dura M Enrichment of endoplasmic reticulum with cholesterol inhibits sarcoplasmic-endoplasmic reticulum calcium ATPase-2b activity in parallel with increased order of membrane lipids: implications for depletion of endoplasmic reticulum calcium stores and apoptosis in cholesterol-loaded macrophages. J Biol Chem (2004) 279:37030–9. 10.1074/jbc.M405195200 15215242

[69] Fu S Yang L Li P Hofmann O Dicker L Hide W Aberrant lipid metabolism disrupts calcium homeostasis causing liver endoplasmic reticulum stress in obesity. Nature (2011) 473:528–31. 10.1038/nature09968 21532591 PMC3102791

[70] Cunha DA Hekerman P Ladriere L Bazarra-Castro A Ortis F Wakeham MC Initiation and execution of lipotoxic ER stress in pancreatic beta-cells. J Cell Sci (2008) 121:2308–18. 10.1242/jcs.026062 18559892 PMC3675788

[71] Sbiera S Leich E Liebisch G Sbiera I Schirbel A Wiemer L Mitotane inhibits sterol-O-acyl transferase 1 triggering lipid-mediated endoplasmic reticulum stress and apoptosis in adrenocortical carcinoma cells. Endocrinology (2015) 156:3895–908. 10.1210/en.2015-1367 26305886

[72] Li HY Appelbaum FR Willman CL Zager RA Banker DE. Cholesterol-modulating agents kill acute myeloid leukemia cells and sensitize them to therapeutics by blocking adaptive cholesterol responses. Blood (2003) 101:3628–34. 10.1182/blood-2002-07-2283 12506040

[73] Baggetto LG Clottes E Vial C. Low mitochondrial proton leak due to high membrane cholesterol content and cytosolic creatine kinase as two features of the deviant bioenergetics of Ehrlich and AS30-D tumor cells. Cancer Res (1992) 52:4935–41. 1516050

[74] Lucken-Ardjomande S Montessuit S Martinou JC Bax activation and stress-induced apoptosis delayed by the accumulation of cholesterol in mitochondrial membranes. Cell Death Differ (2008) 15:484–93. 10.1038/sj.cdd.4402280 18084240

[75] Lucken-Ardjomande S Montessuit S Martinou JC Contributions to Bax insertion and oligomerization of lipids of the mitochondrial outer membrane. Cell Death Differ (2008) 15:929–37. 10.1038/cdd.2008.9 18259190

[76] Ettinger SL Sobel R Whitmore TG Akbari M Bradley DR Gleave ME Dysregulation of sterol response element-binding proteins and downstream effectors in prostate cancer during progression to androgen independence. Cancer Res (2004) 64:2212–21. 10.1158/0008-5472.CAN-2148-2 15026365

[77] Zaidi N Lupien L Kuemmerle NB Kinlaw WB Swinnen JV Smans K. Lipogenesis and lipolysis: the pathways exploited by the cancer cells to acquire fatty acids. Prog Lipid Res (2013) 52:585–9. 10.1016/j.plipres.2013.08.005 24001676 PMC4002264

[78] Howatson AF Ham AW Electron microscope study of sections of two rat liver tumors. Cancer Res (1955) 15:62–9. 13231076

[79] Aisenberg AC Studies on normal and neoplastic mitochondria. I. Respiration. Cancer Res (1961) 21:295–303. 13681828

[80] Hruban Z Mochizuki Y Slesers A Morris HP A comparative study of cellular organelles of Morris hepatomas. Cancer Res (1972) 32:853–67. 4335504

[81] Novikoff AB A transplantable rat liver tumor induced by 4-dimethylaminoazobenzene. Cancer Res (1957) 17:1010–27. 13489702

[82] Reynafarje B Lehninger AL. Ca2+ transport by mitochondria from L1210 mouse ascites tumor cells. Proc Natl Acad Sci U S A (1973) 70:1744–8. 10.1073/pnas.70.6.1744 4515933 PMC433586

[83] Thorne RF Bygrave FL Energy-linked functions of tightly coupled mitochondria isolated from Ehrlich ascites tumor cells. Cancer Res (1973) 33:2562–7. 4270636

[84] Tosatto A Sommaggio R Kummerow C Bentham RB Blacker TS Berecz T The mitochondrial calcium uniporter regulates breast cancer progression via HIF-1alpha. EMBO Mol Med (2016) 8:569–85. 10.15252/emmm.201606255 27138568 PMC4864890

[85] Marchi S Lupini L Patergnani S Rimessi A Missiroli S Bonora M Downregulation of the mitochondrial calcium uniporter by cancer-related miR-25. Curr Biol (2013) 23:58–63. 10.1016/j.cub.2012.11.026 23246404 PMC3540261

[86] Cardenas C Muller M McNeal A Lovy A Jana F Bustos G Selective vulnerability of cancer cells by inhibition of Ca(2+) transfer from endoplasmic reticulum to mitochondria. Cell Rep (2016) 14:2313–24. 10.1016/j.celrep.2016.02.030 26947070 PMC4794382

[87] Giorgi C Bonora M Sorrentino G Missiroli S Poletti F Suski JM p53 at the endoplasmic reticulum regulates apoptosis in a Ca2+-dependent manner. Proc Natl Acad Sci U S A (2015) 112:1779–84. 10.1073/pnas.1410723112 25624484 PMC4330769

[88] Bononi A Bonora M Marchi S Missiroli S Poletti F Giorgi C Identification of PTEN at the ER and MAMs and its regulation of Ca(2+) signaling and apoptosis in a protein phosphatase-dependent manner. Cell Death Differ (2013) 20:1631–43. 10.1038/cdd.2013.77 23811847 PMC3824603

[89] Betz C Stracka D Prescianotto-Baschong C Frieden M Demaurex N Hall MN. Feature article: mTOR complex 2-Akt signaling at mitochondria-associated endoplasmic reticulum membranes (MAM) regulates mitochondrial physiology. Proc Natl Acad Sci U S A (2013) 110:12526–34. 10.1073/pnas.1302455110 23852728 PMC3732980

[90] Hedgepeth SC Garcia MI Wagner LE II Rodriguez AM Chintapalli SV Snyder RR The BRCA1 tumor suppressor binds to inositol 1,4,5-trisphosphate receptors to stimulate apoptotic calcium release. J Biol Chem (2015) 290:7304–13. 10.1074/jbc.M114.611186 25645916 PMC4358148

[91] Giorgi C Ito K Lin HK Santangelo C Wieckowski MR Lebiedzinska M PML regulates apoptosis at endoplasmic reticulum by modulating calcium release. Science (2010) 330:1247–51. 10.1126/science.1189157 21030605 PMC3017677

[92] Missiroli S Bonora M Patergnani S Poletti F Perrone M Gafa R PML at mitochondria-associated membranes is critical for the repression of autophagy and cancer development. Cell Rep (2016) 16:2415–27. 10.1016/j.celrep.2016.07.082 27545895 PMC5011426

[93] Giorgi C Bonora M Missiroli S Morganti C Morciano G Wieckowski MR Alterations in mitochondrial and endoplasmic reticulum signaling by p53 mutants. Front Oncol (2016) 6:42. 10.3389/fonc.2016.00042 26942128 PMC4766755

[94] Vaseva AV Marchenko ND Ji K Tsirka SE Holzmann S Moll UM. p53 opens the mitochondrial permeability transition pore to trigger necrosis. Cell (2012) 149:1536–48. 10.1016/j.cell.2012.05.014 22726440 PMC3383624

[95] Alexandrova EM Yallowitz AR Li D Xu S Schulz R Proia DA Improving survival by exploiting tumour dependence on stabilized mutant p53 for treatment. Nature (2015) 523:352–6. 10.1038/nature14430 26009011 PMC4506213

[96] Bergeaud M Mathieu L Guillaume A Moll UM Mignotte B Le Floch N Mitochondrial p53 mediates a transcription-independent regulation of cell respiration and interacts with the mitochondrial F(1)F0-ATP synthase. Cell Cycle (2013) 12:2781–93. 10.4161/cc.25870 23966169 PMC3899192

[97] Alavian KN Beutner G Lazrove E Sacchetti S Park HA Licznerski P An uncoupling channel within the c-subunit ring of the F1FO ATP synthase is the mitochondrial permeability transition pore. Proc Natl Acad Sci U S A (2014) 111:10580–5. 10.1073/pnas.1401591111 24979777 PMC4115574

[98] Bonora M Bononi A De Marchi E Giorgi C Lebiedzinska M Marchi S Role of the c subunit of the FO ATP synthase in mitochondrial permeability transition. Cell Cycle (2013) 12:674–83. 10.4161/cc.23599 23343770 PMC3594268

[99] Giorgio V von Stockum S Antoniel M Fabbro A Fogolari F Forte M Dimers of mitochondrial ATP synthase form the permeability transition pore. Proc Natl Acad Sci U S A (2013) 110:5887–92. 10.1073/pnas.1217823110 23530243 PMC3625323

[100] Beutner G Ruck A Riede B Brdiczka D. Complexes between porin, hexokinase, mitochondrial creatine kinase and adenylate translocator display properties of the permeability transition pore. Implication for regulation of permeability transition by the kinases. Biochim Biophys Acta (1998) 1368:7–18. 10.1016/S0005-2736(97)00175-2 9459579

[101] Pastorino JG Hoek JB. Regulation of hexokinase binding to VDAC. J Bioenerg Biomembr (2008) 40:171–82. 10.1007/s10863-008-9148-8 18683036 PMC2662512

[102] Wang L Xiong H Wu F Zhang Y Wang J Zhao L Hexokinase 2-mediated Warburg effect is required for PTEN- and p53-deficiency-driven prostate cancer growth. Cell Rep (2014) 8:1461–74. 10.1016/j.celrep.2014.07.053 25176644 PMC4360961

[103] Danese A Patergnani S Bonora M Wieckowski MR Previati M Giorgi C Calcium regulates cell death in cancer: roles of the mitochondria and mitochondria-associated membranes (MAMs). Biochim Biophys Acta (2017). 10.1016/j.bbabio.2017.01.003 28087257

[104] Missiroli S Danese A Iannitti T Patergnani S Perrone M Previati M Endoplasmic reticulum-mitochondria Ca2+ crosstalk in the control of the tumor cell fate. Biochim Biophys Acta (2017) 1864:858–64. 10.1016/j.bbamcr.2016.12.024 28064002

[105] Herrera-Cruz MS Simmen T. Of yeast, mice and men: MAMs come in two flavors. Biol Direct (2017) 12:3. 10.1186/s13062-017-0174-5 28122638 PMC5267431

[106] Kornmann B Currie E Collins SR Schuldiner M Nunnari J Weissman JS An ER-mitochondria tethering complex revealed by a synthetic biology screen. Science (2009) 325:477–81. 10.1126/science.1175088 19556461 PMC2933203

[107] Lahiri S Chao JT Tavassoli S Wong AK Choudhary V Young BP A conserved endoplasmic reticulum membrane protein complex (EMC) facilitates phospholipid transfer from the ER to mitochondria. PLoS Biol (2014) 12:e1001969. 10.1371/journal.pbio.1001969 25313861 PMC4196738

[108] Filadi R Theurey P Pizzo P. The endoplasmic reticulum-mitochondria coupling in health and disease: molecules, functions and significance. Cell Calcium (2017) 62:1–15. 10.1016/j.ceca.2017.01.003 28108029

[109] Zorzano A Liesa M Sebastian D Segales J Palacin M. Mitochondrial fusion proteins: dual regulators of morphology and metabolism. Semin Cell Dev Biol (2010) 21:566–74. 10.1016/j.semcdb.2010.01.002 20079867

[110] Weaver D Eisner V Liu X Varnai P Hunyady L Gross A Distribution and apoptotic function of outer membrane proteins depend on mitochondrial fusion. Mol Cell (2014) 54:870–8. 10.1016/j.molcel.2014.03.048 24813948 PMC4363113

[111] Li L Gao G Shankar J Joshi B Foster LJ Nabi IR. p38 MAP kinase-dependent phosphorylation of the Gp78 E3 ubiquitin ligase controls ER-mitochondria association and mitochondria motility. Mol Biol Cell (2015) 26:3828–40. 10.1091/mbc.E15-02-0120 26337390 PMC4626067

[112] Wang PT Garcin PO Fu M Masoudi M St-Pierre P Pante N Distinct mechanisms controlling rough and smooth endoplasmic reticulum contacts with mitochondria. J Cell Sci (2015) 128:2759–65. 10.1242/jcs.171132 26065430

[113] de Brito OM Scorrano L. Mitofusin 2 tethers endoplasmic reticulum to mitochondria. Nature (2008) 456:605–10. 10.1038/nature07534 19052620

[114] Alford SC Ding Y Simmen T Campbell RE. Dimerization-dependent green and yellow fluorescent proteins. ACS Synth Biol (2012) 1:569–75. 10.1021/sb300050j 23656278 PMC3653836

[115] Naon D Zaninello M Giacomello M Varanita T Grespi F Lakshminaranayan S Critical reappraisal confirms that mitofusin 2 is an endoplasmic reticulum-mitochondria tether. Proc Natl Acad Sci U S A (2016) 113(40):11249–54. 10.1073/pnas.1606786113 27647893 PMC5056088

[116] Schneeberger M Dietrich MO Sebastian D Imbernon M Castano C Garcia A Mitofusin 2 in POMC neurons connects ER stress with leptin resistance and energy imbalance. Cell (2013) 155:172–87. 10.1016/j.cell.2013.09.003 24074867 PMC3839088

[117] Munoz JP Ivanova S Sanchez-Wandelmer J Martinez-Cristobal P Noguera E Sancho A Mfn2 modulates the UPR and mitochondrial function via repression of PERK. EMBO J (2013) 32:2348–61. 10.1038/emboj.2013.168 23921556 PMC3770335

[118] Cosson P Marchetti A Ravazzola M Orci L. Mitofusin-2 independent juxtaposition of endoplasmic reticulum and mitochondria: an ultrastructural study. PLoS One (2012) 7:e46293. 10.1371/journal.pone.0046293 23029466 PMC3460865

[119] Filadi R Greotti E Turacchio G Luini A Pozzan T Pizzo P. Mitofusin 2 ablation increases endoplasmic reticulum-mitochondria coupling. Proc Natl Acad Sci U S A (2015) 112:E2174–81. 10.1073/pnas.1504880112 25870285 PMC4418914

[120] Bravo R Vicencio JM Parra V Troncoso R Munoz JP Bui M Increased ER-mitochondrial coupling promotes mitochondrial respiration and bioenergetics during early phases of ER stress. J Cell Sci (2011) 124:2143–52. 10.1242/jcs.080762 21628424 PMC3113668

[121] Csordas G Renken C Varnai P Walter L Weaver D Buttle KF Structural and functional features and significance of the physical linkage between ER and mitochondria. J Cell Biol (2006) 174:915–21. 10.1083/jcb.200604016 16982799 PMC2064383

[122] Guo X Chen KH Guo Y Liao H Tang J Xiao RP. Mitofusin 2 triggers vascular smooth muscle cell apoptosis via mitochondrial death pathway. Circ Res (2007) 101:1113–22. 10.1161/CIRCRESAHA.107.157644 17901359

[123] Wan-Xin T Tian-Lei C Ben W Wei-Hua W Ping F. Effect of mitofusin 2 overexpression on the proliferation and apoptosis of high-glucose-induced rat glomerular mesangial cells. J Nephrol (2012) 25:1023–30. 10.5301/jn.5000089 22322822

[124] Wang W Xie Q Zhou X Yao J Zhu X Huang P Mitofusin-2 triggers mitochondria Ca2+ influx from the endoplasmic reticulum to induce apoptosis in hepatocellular carcinoma cells. Cancer Lett (2015) 358:47–58. 10.1016/j.canlet.2014.12.025 25541060

[125] Wu Y Zhou D Xu X Zhao X Huang P Zhou X Clinical significance of mitofusin-2 and its signaling pathways in hepatocellular carcinoma. World J Surg Oncol (2016) 14:179. 10.1186/s12957-016-0922-5 27389277 PMC4936233

[126] Zhou X Zhang L Zheng B Yan Y Zhang Y Xie H MicroRNA-761 is upregulated in hepatocellular carcinoma and regulates tumorigenesis by targeting mitofusin-2. Cancer Sci (2016) 107:424–32. 10.1111/cas.12904 26845057 PMC4832850

[127] Ma LI Chang Y Yu L He W Liu Y. Pro-apoptotic and anti-proliferative effects of mitofusin-2 via PI3K/Akt signaling in breast cancer cells. Oncol Lett (2015) 10:3816–22. 10.3892/ol.2015.3748 26788214 PMC4665343

[128] Ding Y Gao H Zhao L Wang X Zheng M. Mitofusin 2-deficiency suppresses cell proliferation through disturbance of autophagy. PLoS One (2015) 10:e0121328. 10.1371/journal.pone.0121328 25781899 PMC4363693

[129] Kottgen M Benzing T Simmen T Tauber R Buchholz B Feliciangeli S Trafficking of TRPP2 by PACS proteins represents a novel mechanism of ion channel regulation. EMBO J (2005) 24:705–16. 10.1038/sj.emboj.7600566 15692563 PMC549624

[130] Simmen T Aslan JE Blagoveshchenskaya AD Thomas L Wan L Xiang Y PACS-2 controls endoplasmic reticulum-mitochondria communication and Bid-mediated apoptosis. EMBO J (2005) 24:717–29. 10.1038/sj.emboj.7600637 15692567 PMC549619

[131] Youker RT Shinde U Day R Thomas G. At the crossroads of homoeostasis and disease: roles of the PACS proteins in membrane traffic and apoptosis. Biochem J (2009) 421:1–15. 10.1042/BJ20081016 19505291 PMC4303049

[132] Thomas G Aslan JE Thomas L Shinde P Shinde U Simmen T. Caught in the act - protein adaptation and the expanding roles of the PACS proteins in tissue homeostasis and disease. J Cell Sci (2017). 10.1242/jcs.199463 28476937 PMC5482974

[133] Raturi A Ortiz-Sandoval C Simmen T Redox dependence of endoplasmic reticulum (ER) Ca(2)(+) signaling. Histol Histopathol (2014) 29:543–52. 10.14670/HH-29.10.543 24197491

[134] Aslan JE You H Williamson DM Endig J Youker RT Thomas L Akt and 14-3-3 control a PACS-2 homeostatic switch that integrates membrane traffic with TRAIL-induced apoptosis. Mol Cell (2009) 34:497–509. 10.1016/j.molcel.2009.04.011 19481529 PMC2744858

[135] Boehning D Patterson RL Sedaghat L Glebova NO Kurosaki T Snyder SH. Cytochrome c binds to inositol (1,4,5) trisphosphate receptors, amplifying calcium-dependent apoptosis. Nat Cell Biol (2003) 5:1051–61. 10.1038/ncb1063 14608362

[136] Anderson GR Brenner BM Swede H Chen N Henry WM Conroy JM Intrachromosomal genomic instability in human sporadic colorectal cancer measured by genome-wide allelotyping and inter-(simple sequence repeat) PCR. Cancer Res (2001) 61:8274–83. 11719460

[137] Li BQ Huang T Zhang J Zhang N Huang GH Liu L An ensemble prognostic model for colorectal cancer. PLoS One (2013) 8:e63494. 10.1371/journal.pone.0063494 23658834 PMC3642113

[138] Voeltz GK Prinz WA Shibata Y Rist JM Rapoport TA. A class of membrane proteins shaping the tubular endoplasmic reticulum. Cell (2006) 124:573–86. 10.1016/j.cell.2005.11.047 16469703

[139] Teng FY Tang BL. Cell autonomous function of Nogo and reticulons: the emerging story at the endoplasmic reticulum. J Cell Physiol (2008) 216:303–8. 10.1002/jcp.21434 18330888

[140] Belmont PJ Tadimalla A Chen WJ Martindale JJ Thuerauf DJ Marcinko M Coordination of growth and endoplasmic reticulum stress signaling by regulator of calcineurin 1 (RCAN1), a novel ATF6-inducible gene. J Biol Chem (2008) 283:14012–21. 10.1074/jbc.M709776200 18319259 PMC2376224

[141] Sutendra G Dromparis P Wright P Bonnet S Haromy A Hao Z The role of Nogo and the mitochondria-endoplasmic reticulum unit in pulmonary hypertension. Sci Transl Med (2011) 3:88ra55. 10.1126/scitranslmed.3002194 21697531 PMC3744110

[142] Reali V Mehdawy B Nardacci R Filomeni G Risuglia A Rossin F Reticulon protein-1C is a key component of MAMs. Biochim Biophys Acta (2015) 1853:733–45. 10.1016/j.bbamcr.2014.12.031 25573430

[143] Jozsef L Tashiro K Kuo A Park EJ Skoura A Albinsson S Reticulon 4 is necessary for endoplasmic reticulum tubulation, STIM1-Orai1 coupling, and store-operated calcium entry. J Biol Chem (2014) 289:9380–95. 10.1074/jbc.M114.548602 24558039 PMC3969502

[144] Li Q Qi B Oka K Shimakage M Yoshioka N Inoue H Link of a new type of apoptosis-inducing gene ASY/Nogo-B to human cancer. Oncogene (2001) 20:3929–36. 10.1038/sj.onc.1204536 11494121

[145] Shimakage M Inoue N Ohshima K Kawahara K Oka T Yasui K Down-regulation of ASY/Nogo transcription associated with progression of adult T-cell leukemia/lymphoma. Int J Cancer (2006) 119:1648–53. 10.1002/ijc.22011 16646068

[146] Calik J Pula B Piotrowska A Wojnar A Witkiewicz W Grzegrzolka J Prognostic significance of NOGO-A/B and NOGO-B receptor expression in malignant melanoma – a preliminary study. Anticancer Res (2016) 36:3401–7. 27354599

[147] Ahn DG Sharif T Chisholm K Pinto DM Gujar SA Lee PW. Ras transformation results in cleavage of reticulon protein Nogo-B that is associated with impairment of IFN response. Cell Cycle (2015) 14:2301–10. 10.1080/15384101.2015.1044187 25946643 PMC4614670

[148] Jin SG Ryu HH Li SY Li CH Lim SH Jang WY Nogo-A inhibits the migration and invasion of human malignant glioma U87MG cells. Oncol Rep (2016) 35:3395–402. 10.3892/or.2016.4737 27109183

[149] Hatakeyama J Wald JH Rafidi H Cuevas A Sweeney C Carraway KL III. The ER structural protein Rtn4A stabilizes and enhances signaling through the receptor tyrosine kinase ErbB3. Sci Signal (2016) 9:ra65. 10.1126/scisignal.aaf1604 27353365 PMC5554593

[150] Verfaillie T Rubio N Garg AD Bultynck G Rizzuto R Decuypere JP PERK is required at the ER-mitochondrial contact sites to convey apoptosis after ROS-based ER stress. Cell Death Differ (2012) 19:1880–91. 10.1038/cdd.2012.74 22705852 PMC3469056

[151] Hetz C. The unfolded protein response: controlling cell fate decisions under ER stress and beyond. Nat Rev Mol Cell Biol (2012) 13:89–102. 10.1038/nrm3270 22251901

[152] van Vliet AR Agostinis P. When under pressure, get closer: PERKing up membrane contact sites during ER stress. Biochem Soc Trans (2016) 44:499–504. 10.1042/BST20150272 27068961

[153] van Vliet AR Giordano F Gerlo S Segura I Van Eygen S Molenberghs G The ER stress sensor PERK coordinates ER-plasma membrane contact site formation through interaction with filamin-A and F-actin remodeling. Mol Cell (2017) 65:885–99.e6. 10.1016/j.molcel.2017.01.020 28238652

[154] Fels DR Koumenis C. The PERK/eIF2alpha/ATF4 module of the UPR in hypoxia resistance and tumor growth. Cancer Biol Ther (2006) 5:723–8. 10.4161/cbt.5.7.2967 16861899

[155] Harding HP Zhang Y Zeng H Novoa I Lu PD Calfon M An integrated stress response regulates amino acid metabolism and resistance to oxidative stress. Mol Cell (2003) 11:619–33. 10.1016/S1097-2765(03)00105-9 12667446

[156] Bu Y Diehl JA. PERK integrates oncogenic signaling and cell survival during cancer development. J Cell Physiol (2016) 231:2088–96. 10.1002/jcp.25336 26864318 PMC4912452

[157] Pytel D Gao Y Mackiewicz K Katlinskaya YV Staschke KA Paredes MC PERK is a haploinsufficient tumor suppressor: gene dose determines tumor-suppressive versus tumor promoting properties of PERK in melanoma. PLoS Genet (2016) 12:e1006518 10.1371/journal.pgen.1006518 27977682 PMC5207760

[158] Iwasawa R Mahul-Mellier AL Datler C Pazarentzos E Grimm S. Fis1 and Bap31 bridge the mitochondria-ER interface to establish a platform for apoptosis induction. EMBO J (2011) 30:556–68. 10.1038/emboj.2010.346 21183955 PMC3034017

[159] Breckenridge DG Stojanovic M Marcellus RC Shore GC. Caspase cleavage product of BAP31 induces mitochondrial fission through endoplasmic reticulum calcium signals, enhancing cytochrome c release to the cytosol. J Cell Biol (2003) 160:1115–27. 10.1083/jcb.200212059 12668660 PMC2172754

[160] Yu S Wang F Fan L Wei Y Li H Sun Y BAP31, a promising target for the immunotherapy of malignant melanomas. J Exp Clin Cancer Res (2015) 34:36. 10.1186/s13046-015-0153-6 25903101 PMC4405826

[161] Wang K Long B Jiao JQ Wang JX Liu JP Li Q miR-484 regulates mitochondrial network through targeting Fis1. Nat Commun (2012) 3:781. 10.1038/ncomms1770 22510686

[162] Szabadkai G Bianchi K Varnai P De Stefani D Wieckowski MR Cavagna D Chaperone-mediated coupling of endoplasmic reticulum and mitochondrial Ca2+ channels. J Cell Biol (2006) 175:901–11. 10.1083/jcb.200608073 17178908 PMC2064700

[163] Rapizzi E Pinton P Szabadkai G Wieckowski MR Vandecasteele G Baird G Recombinant expression of the voltage-dependent anion channel enhances the transfer of Ca2+ microdomains to mitochondria. J Cell Biol (2002) 159:613–24. 10.1083/jcb.200205091 12438411 PMC2173108

[164] Cardenas C Muller M McNeal A Lovy A Jana F Bustos G Selective vulnerability of cancer cells by inhibition of Ca(2+) transfer from endoplasmic reticulum to mitochondria. Cell Rep (2016) 15:219–20. 10.1016/j.celrep.2016.03.045 27050774

[165] Shoshan-Barmatz V Ben-Hail D Admoni L Krelin Y Tripathi SS. The mitochondrial voltage-dependent anion channel 1 in tumor cells. Biochim Biophys Acta (2015) 1848:2547–75. 10.1016/j.bbamem.2014.10.040 25448878

[166] Pernemalm M De Petris L Branca RM Forshed J Kanter L Soria JC Quantitative proteomics profiling of primary lung adenocarcinoma tumors reveals functional perturbations in tumor metabolism. J Proteome Res (2013) 12:3934–43. 10.1021/pr4002096 23902561

[167] Wadhwa R Kaul SC Ikawa Y Sugimoto Y. Identification of a novel member of mouse hsp70 family. Its association with cellular mortal phenotype. J Biol Chem (1993) 268:6615–21. 8454632

[168] Yi X Luk JM Lee NP Peng J Leng X Guan XY Association of mortalin (HSPA9) with liver cancer metastasis and prediction for early tumor recurrence. Mol Cell Proteomics (2008) 7:315–25. 10.1074/mcp.M700116-MCP200 17934217

[169] Wadhwa R Takano S Kaur K Deocaris CC Pereira-Smith OM Reddel RR Upregulation of mortalin/mthsp70/Grp75 contributes to human carcinogenesis. Int J Cancer (2006) 118:2973–80. 10.1002/ijc.21773 16425258

[170] Merrick BA He C Witcher LL Patterson RM Reid JJ Pence-Pawlowski PM HSP binding and mitochondrial localization of p53 protein in human HT1080 and mouse C3H10T1/2 cell lines. Biochim Biophys Acta (1996) 1297:57–68. 10.1016/0167-4838(96)00089-1 8841381

[171] De Vos KJ Morotz GM Stoica R Tudor EL Lau KF Ackerley S VAPB interacts with the mitochondrial protein PTPIP51 to regulate calcium homeostasis. Hum Mol Genet (2012) 21:1299–311. 10.1093/hmg/ddr559 22131369 PMC3284118

[172] Petri MK Brobeil A Planz J Brauninger A Gattenlohner S Nestler U PTPIP51 levels in glioblastoma cells depend on inhibition of the EGF-receptor. J Neurooncol (2015) 123:15–25. 10.1007/s11060-015-1763-8 25862004

[173] Petri MK Koch P Stenzinger A Kuchelmeister K Nestler U Paradowska A PTPIP51, a positive modulator of the MAPK/Erk pathway, is upregulated in glioblastoma and interacts with 14-3-3beta and PTP1B in situ. Histol Histopathol (2011) 26:1531–43. 21972092 10.14670/HH-26.1531

[174] Rao M Song W Jiang A Shyr Y Lev S Greenstein D VAMP-associated protein B (VAPB) promotes breast tumor growth by modulation of Akt activity. PLoS One (2012) 7:e46281. 10.1371/journal.pone.0046281 23049696 PMC3462209

[175] Stoica R De Vos KJ Paillusson S Mueller S Sancho RM Lau KF ER-mitochondria associations are regulated by the VAPB-PTPIP51 interaction and are disrupted by ALS/FTD-associated TDP-43. Nat Commun (2014) 5:3996. 10.1038/ncomms4996 24893131 PMC4046113

[176] Stoica R Paillusson S Gomez-Suaga P Mitchell JC Lau DH Gray EH ALS/FTD-associated FUS activates GSK-3beta to disrupt the VAPB-PTPIP51 interaction and ER-mitochondria associations. EMBO Rep (2016) 17:1326–42. 10.15252/embr.201541726 27418313 PMC5007559

[177] Raturi A Gutierrez T Ortiz-Sandoval C Ruangkittisakul A Herrera-Cruz MS Rockley JP TMX1 determines cancer cell metabolism as a thiol-based modulator of ER-mitochondria Ca2+ flux. J Cell Biol (2016) 214:433–44. 10.1083/jcb.201512077 27502484 PMC4987292

